# WISARD: workbench for integrated superfast association studies for related datasets

**DOI:** 10.1186/s12920-018-0345-y

**Published:** 2018-04-20

**Authors:** Sungyoung Lee, Sungkyoung Choi, Dandi Qiao, Michael Cho, Edwin K. Silverman, Taesung Park, Sungho Won

**Affiliations:** 1 Interdisciplinary Program in Bioinformatics, Seoul National University, Seoul, South Korea; 2 Department of Pharmacology, Yonsei University College of Medicine, Seoul, South Korea; 3 Channing Division of Network Medicine, Department of Medicine, Brigham and Women’s Hospital and Harvard Medical School, Boston, MA USA; 4 Division of Pulmonary and Critical Care Medicine, Brigham and Women’s Hospital, Boston, MA USA; 5 Department of Statistics, Seoul National University, 1 Kwanak-ro, Kwanak-gu, Seoul, 151-742 South Korea; 6 Department of Public Health Sciences, Graduate School of Public Health, Seoul National University, 1 Kwanak-ro, Kwanak-gu, Seoul, 151-742 South Korea; 7 Institute of Health and Environment, Seoul National University, Seoul, South Korea

**Keywords:** Family-based design, Genome-wide association analyses, Next generation sequencing, Multi-threaded analyses, Related samples

## Abstract

**Background:**

A Mendelian transmission produces phenotypic and genetic relatedness between family members, giving family-based analytical methods an important role in genetic epidemiological studies—from heritability estimations to genetic association analyses. With the advance in genotyping technologies, whole-genome sequence data can be utilized for genetic epidemiological studies, and family-based samples may become more useful for detecting de novo mutations. However, genetic analyses employing family-based samples usually suffer from the complexity of the computational/statistical algorithms, and certain types of family designs, such as incorporating data from extended families, have rarely been used.

**Results:**

We present a Workbench for Integrated Superfast Association studies for Related Data (WISARD) programmed in C/C++. WISARD enables the fast and a comprehensive analysis of SNP-chip and next-generation sequencing data on extended families, with applications from designing genetic studies to summarizing analysis results. In addition, WISARD can automatically be run in a fully multithreaded manner, and the integration of R software for visualization makes it more accessible to non-experts.

**Conclusions:**

Comparison with existing toolsets showed that WISARD is computationally suitable for integrated analysis of related subjects, and demonstrated that WISARD outperforms existing toolsets. WISARD has also been successfully utilized to analyze the large-scale massive sequencing dataset of chronic obstructive pulmonary disease data (COPD), and we identified multiple genes associated with COPD, which demonstrates its practical value.

**Electronic supplementary material:**

The online version of this article (10.1186/s12920-018-0345-y) contains supplementary material, which is available to authorized users.

## Background

Family-based samples have different properties from population-based samples because of Mendelian transmission, and this well-known feature has allowed family-based designs to play a key role in genetic epidemiology from the very beginning of genetic analysis. For instance, phenotypic correlations between family members enable the estimation of heritability via a linear mixed effects model [1], and linkage analyses have helped identify the disease-causing loci using a few large families [2–5]. Recently, rare variants have been recognized as a main source for the so-called missing heritability [6], and the importance of family-based designs has been repeatedly stressed for analyses with sequence data because of genetic homogeneity between family members [7].

Furthermore, in the presence of population substructure, statistical methods for association analysis with population-based samples are often similar to those for family-based samples. The presence of population substructure generates correlations between population-based samples, and the magnitude of the correlation can be substantial for phenotypes with a large polygenic effect. For instance, around 30% of the phenotypic variance of height is captured by the genetic relationship matrix (GRM) [8] and the linear mixed effects model can be used to take into account the correlations between subjects. For quantitative phenotypes, a number of methods with high computational efficiency have recently been introduced [9, 10], and have been successfully applied to genome-wide association studies [11–13]. For dichotomous phenotypes, the nonlinear models might be considered to be a reasonable and appropriate approach. However, generalized linear mixed effects models that use maximum likelihood for estimation and approximations to avoid numerical integration have a serious bias introduced by the approximation [14, 15]. In order to overcome this issue, score statistics such as FBAT [16] and MQLS [17] have been proposed as alternatives.

**Fig. 1:**
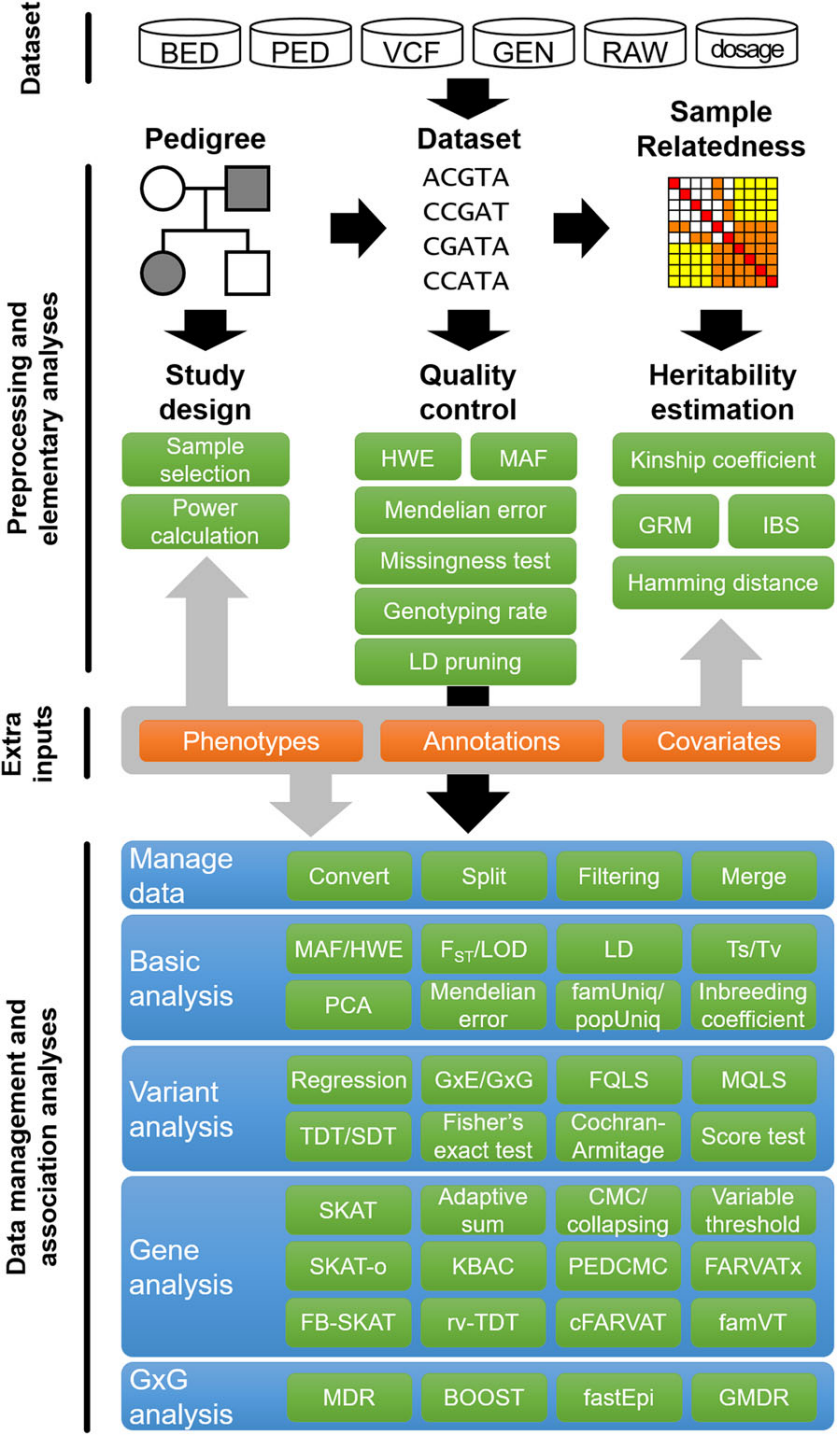
Lists of tasks supported by WISARD. *famUniq* (Family-unique variants), *popUniq* (P opulation-unique variants), *fastEpi* (Fast-epistasis method), *FQLS* (Family QLS method), *inbCoef* (Inbreeding coefficients), *MFQLS* (Multivariate FQLS)

However, in spite of such improvement, there is no integrated toolset for an analysis of large-scale family-based samples, and statistical analysis has often suffered from computational intensity. In this paper, we introduce a Workbench for Integrated Superfast Analyses for Related Data (WISARD), which is thoroughly optimized for analysis in a multi-core system and has comprehensive features for various analyses. Furthermore we propose two novel methods for rare variant association analysis with related samples, cFARVAT and famVT. WISARD features three major functionalities: data management, quality control (QC), and association analysis. WISARD provides various tasks for large-scale genetic data management such as retrieval, conversion, split and merging of datasets with various formats. For extended families, the family-based imputation performed by WISARD is useful for handling missing genotypes. Second, genotype quality for each variant or each subject can be evaluated using several statistics, and samples and variants can be filtered based on quality scores. Third, WISARD provides useful functions for association analyses ranging from heritability estimations to joint association analysis with multiple genotypes and phenotypes. With the integration of R, WISARD enables longitudinal data analysis, and the graphical summarization of analysis results. A list of tasks supported by WISARD with family-based samples is shown in Fig. 1.

To demonstrate the performance and practicality of WISARD, we compared its performance with existing toolsets by using genetic analysis workshop (GAW) 18 dataset, and statistical powers of two new methods for rare variant association analysis were evaluated with GAW17 dataset. Moreover, we also analyzed the Boston Early Onset Chronic Obstructive Pulmonary Disease (EOCOPD) dataset. We found that WISARD outperforms existing toolsets. These results illustrate the practical value of WISARD, and how it can strengthen the analytical power of analysis of large-scale genetic datasets for newcomers.

## Implementation

### Data management

WISARD has many functions related to data management and can simply conduct retrieval, conversion, splitting and merging of a large-scale genetic data with various formats. At the same time, we provide a simple method to impute missing genotypes for typed variants based on the familial relationship and calculate the expected genotypes for untyped subjects. For instance, if the phenotypes of any untyped subjects in each family are available, their genotypes can be imputed from their relatives’ genotypes and then imputed genotypes can be utilized for genetic association analysis. More than 60 functions for data managements are available, providing enormous convenience for analysis. Furthermore 15 different file formats including variant call format (VCF) can be easily converted to other file formats.

### Quality control

Many functions for quality control and summarization measures for both chip-based and sequencing-based data are implemented. The quality control process can be accomplished by many QC measures such as Hardy-Weinberg equilibrium, minor allele frequency (MAF), minor allele count (MAC), genotype missing rate, or Mendelian error rate. In addition, many elementary statistics for genetic datasets are also provided, including Ts/Tv ratio, inbreeding coefficient and Fixation index.

### Statistical analyses

WISARD can perform various association analyses in multi-threaded manner. Statistical analyses implemented in WISARD are listed in Table 1. WISARD can conduct variant-level, gene-gene interaction, and gene-level tests for both dichotomous and quantitative phenotypes. Variant-level tests are usually used for association analysis with common variants and include the linear mixed effects models for quantitative phenotypes [10] and quasi-score tests for the dichotomous phenotype [17, 18]. Extended quasi-score tests for multiple phenotypes and variants [19] are also implemented. For gene-gene interaction, Boolean operation-based screening and testing method [20], and multifactor dimensionality reduction methods [21, 22] are implemented for detecting gene-gene interaction for independent subjects. In addition, gene-level tests are often used for association analysis with rare variants, and cover the combined and multivariate collapsing test [23], variable threshold (VT) method [24], and SKAT [25, 26], etc. In particular, most of gene-level tests were limited to population-based samples, and few approaches available for family-based samples. PEDCMC [27], FARVAT [28], mFARVAT [29], and FARVATx [30] are implemented in WISARD. Furthermore, we provided new statistics, family-based VT and family-based SKAT-o. Both are denoted by famVT and cFARVAT-o in the remainder of this report. Family-based burden and SKAT tests are denoted by cFARVAT-b and cFARVAT-s. Detailed methods for those statistics are provided in Additional file 1: Supplementary text.

**Table 1:** A list of association tests supported by WISARD

Variant types Phenotype	Population-based samples	Family-based samples, or population-based sample under population substructure
Common		
Binary	Cochran-Armitage Logistic regression MDR [22]	TDT/SDT (family-based only) FQLS [44] MFQLS [19] MQLS [17]
Continuous	Linear regression Generalized MDR [21]	Score test for linear mixed model GEMMA [10]
Rare		
Binary	CMC [45] C-alpha [46] KBAC [47] SKAT [25] SKAT-o [26] Weighted-sum test [48]	PEDCMC [27] FARVAT [28] mFARVAT [29] FARVATx [30] FB-SKAT [49] rvTDT [50]
Continuous	VT [24] SKAT [25] SKAT-o [26] Q-test [51]	FARVAT [28] cFARVAT-b / cFARVAT-s / cFARVAT-o famVT

### Implementation

Distinctive feature of WISARD is an implementation of functions for various statistical analyses such as linear mixed effects model, quasi-likelihood approaches. However in spite of their statistical efficiency, parameter estimation for linear mixed models and quasi-likelihood approaches require many matrix related operation and it is computationally very intensive. For instance, genome-wide analyses often take more than a few months if sample size is a few thousands or more. Therefore multiple software have been proposed to improve the computational complexity, and they utilized existing C/C++ libraries for matrix calculation such as EIGEN (http://eigen.tuxfamily.org), and LAPACK (http://www.netlib.org/lapack/). We developed our own C/C++ library for matrix operations, and its computational efficiency improves the computational time of WISARD. Implemented C/C++ library has four different property, compared to existing software; (1) row-wise matrix access, (2) efficient use of symmetric matrix, (3) application of Single-Instruction-Multiple-Data (SIMD), and (4) sweep-operator. Detailed explanation is provided in Additional file 1: Supplementary text.

## Methods

### Comparison of computational efficiency with GAW18 datasets

Computational efficiency was compared with GAW18 simulation dataset. GAW18 dataset has sequences of odd numbered chromosomes for 464 subjects from 20 extended Mexican-American families, and a set of 200 replicated phenotypes were generated from real genotypes. We considered continuous phenotype Q1. We considered variants whose *P* values for HWE are less than 10^− 8^, call rates are larger than 0.95 and Mendelian error rates are less than 0.01. Subjects whose call rates are less than 0.95 and Mendelian error rates are larger than 0.01 were excluded. For performance comparison, we considered variant-level analyses, gene-level analyses and calculating GRM and identity-by-state (IBS) matrix. For gene-level tests, we consider only rare variants whose MAFs are less than 0.05. For GRM, IBS calculation and variant-level tests, we considered variants whose MAFs are larger than 0.05. Multithreaded analyses with 2, 4 and 8 threads were also performed (Additional file 1: Figure S1).

Recently many toolsets for analysis of large-scale sequencing dataset have been proposed, but most of them can analyze only population-based samples. Very few toolsets are available for family-based samples. For instance, PLINK2 is an extension of the well-known toolset for analyses of population-based genetic dataset, PLINK [31], but it is limited to data management and quality controls. In order to demonstrate capability and computational efficiency of WISARD, we consider the recently developed toolsets for large-scale genetic dataset analyses with family-based samples: GEMMA for variant-level analyses [10] and Rvtests for gene-level tests [32]. Rvtests was the most recently developed toolset and provides most comprehensive features for rare variant association analyses. famSKAT [33] is also considered for family-based rare variant association analyses. For common variant association analyses, GEMMA is one of the fastest toolset for linear mixed effects model [10]. FREGAT provides an integrated R framework for gene-level tests with family-based samples. However despite FREGAT provides extensive family-based analyses, it was excluded from the computational performance comparison, since FREGAT is an R package and it runs comparably very slow.

All analyses were performed using a dedicated computing node with two Intel Xeon processors and 128GiB of RAM, and all software were independently executed to minimize any perturbation for checking net performance. Each analysis was executed five times and their mean execution times were compared with their variation.

### Evaluations of new methods with GAW17 datasets

GAW 17 dataset were used to evaluate validity of proposed gene-level tests (famVT and cFARVAT-o). GAW17 is an artificial dataset that consists of a single set of odd-numbered chromosomes generated from 697 subjects from 1000 Genomes Projects, and 200 replicates of simulated phenotypes. We considered continuous phenotype Q1. We considered variants whose *P* values for HWE are less than 10^− 8^, call rates are larger than 0.95 and Mendelian error rates are less than 0.01. Subjects whose call rates are less than 0.95 were excluded. Each variant was annotated with UCSC Genome Browser (genome version GRCh37), and rare variants of which MAFs were less than 0.05 were used to make a gene set file for gene-level tests. To adjust the population substructure, variance-covariance matrix was parameterized with GRM, and variants whose MAFs are larger than 0.05, were used to get GRM matrix.

For evaluation of proposed methods, we estimated the empirical type 1 errors and statistical powers. The empirical type-1 errors were estimated by calculating proportions of non-causal genes whose *P* values are less than several significance levels with 1000 permuted phenotypes. The statistical powers were estimated by using six predefined causal genes of 200 simulated phenotypes in GAW17. Their estimated statistical powers were compared with existing toolsets for family-based analyses: MONSTER [34], famSKAT [33], and famBT, FFBSKAT, MLR in FREGAT [35], as well as the methods for analysis of independent samples: SKAT and CMC.

### Boston Early-onset COPD study dataset

We applied the proposed rare variant association statistics to whole-exome sequencing data from the Boston Early-onset COPD (EOCOPD) Study [36]. Whole exome sequencing was performed at the University of Washington Center for Mendelian Genomics. We utilized the same strategies for quality control of sequencing data by Wang, et al. (2016). Quality control included Mendelian error rates (< 1%), Hardy-Weinberg equilibrium (*P* > 10^− 8^), and average sequencing depth (> 12). Relatedness of subjects was evaluated by comparing the kinship coefficient matrix (KCM) and GRM. Heterozygous/homozygous genotype ratio, Mendelian errors, the proportion of variants in dbSNP, and proportion of nonsynonymous variants were used to identify outliers. After subjects with missing phenotypes or covariates were filtered out and 254 subjects from 49 families were analyzed.

For gene-level rare variant association analyses, we assumed that variants with MAFs less than 0.05 were rare. We then annotated the rare variants to genes with UCSC Genome Browser (genome version GRCh37). We considered genes with at least two rare variants, and 4 or more MAC, and thus 8126 genes that consist of 88,373 rare variants were analyzed. We considered five COPD-related phenotypes: forced expiratory volume in 1 s. prebronchodilator (FEVPRE); forced vital capacity postbronchodilator (FVCPST); forced expiratory flow 25–75% prebronchodilator (DPRF2575); FEVPRE divided by FVCPRE (RATIO); DPRF2575 divided by FVCPRE (F2575RAT). Sex, age, height, and pack-years of cigarette smoking were utilized as covariates. For variance-covariance matrix, we applied both KCM and GRM according to the status of population substructure. Population substructure was not detected and KCM was utilized. The significance level α was set to 0.05, and Bonferroni correction was applied for multiple testing problem.

## Results

### Comparison of available functions

Table 2 shows summary of available functions in WISARD and its functionalities were compared with other toolsets. As was shown in Table 2, PLINK2 [37] lacks association tests for related subjects. GCTA [38] supports single file format and does not support any association analyses. Numbers of filtering functions for WISARD, PLINK2, GCTA and Rvtests are 70, 54, 10 and 17, respectively, and WISARD provides the largest filtering functions. Furthermore WISARD supports regular expression and conditional statement for filtering variants and subjects. Those are helpful for in-depth analysis of the dataset for various purposes. For gene-level association analysis, WISARD supports six types of gene mapping file format: refFlat format, two interval formats and three direct mapping format while other toolsets support only one or two formats. In addition, WISARD has more functions for statistical analyses such as X-chromosome gene-level association analysis with the family-based dataset, FARVATx [30], and allows multi-thread analyses except few analyses such as PCA analysis and heritability with ‘--thread’ option.

**Table 2:** Comparison of available functions for existing toolsets

Category Functions	WISARD	PLINK2	GCTA	FREGAT	Rvtests
Input format					
PED	O	O	X	X	X
Binary PED	O	O	O	O	X
VCF	O	O	X	O	O
Binary VCF	O	O	X	X	X
Dosage	O	O	O	X	X
Others	O	O	X	O	X
Random dataset	O	O	X	X	X
Recode dataset					
PED	O	O	X	X	X
Binary PED	O	O	O	X	X
VCF	O	O	X	X	X
Binary VCF	O	O	X	X	X
Others	O	O	O	X	X
Data manipulation					
# of variant filters	38	27	8	0	11
# of gene filters	4	0	0	0	2
# of subject filters	28	27	2	0	4
Family-specific filters	O	X	X	X	X
VCF-specific filters	O	X	X	X	O
Data merge	O	O	X	X	X
Covariate filters	O	O	X	X	X
Data split	O	O	X	X	X
Distance matrix					
# of input formats	4	1	1	0	1
# of output formats	4	1	1	0	1
# of producible distances	7	2	1	0	4
Data summary					
Variant summary	O	O	X	X	X
Gene summary functions	O	X	X	O	X
Variant-level analysis of unrelated samples					
binary phenotypes	O	O	O	X	O
continuous phenotypes	O	O	O	X	O
multivariate phenotypes	O	O	O	X	O
Gene-level analysis of unrelated samples					
binary phenotypes	O	O	X	O	O
continuous phenotypes	O	O	X	O	O
multivariate phenotypes	O	X	X	X	O
X-chromosome	O	X	X	X	X
Variant-level analysis of related samples					
binary phenotypes	O	X	O	X	O
continuous phenotypes	O	X	O	X	O
multivariate phenotypes	O	X	O	X	O
Gene-level analysis of related samples					
binary phenotypes	O	X	X	O	O
continuous phenotypes	O	X	X	O	O
multivariate phenotypes	O	X	X	X	O
Others features					
Variant-level meta-analysis	O	O	X	X	O
Gene-level meta-analysis	O	X	X	X	O
R connectivity	O	O	X	O	X
Multi-thread analyses	O	O	O	O	O
Programming Language	C/C++	C/C++	C/C++	R	C/C++
# of supported platforms	5	3	1	3	1

### Comparison of computational efficiency with GAW18 datasets

**Fig. 2:**
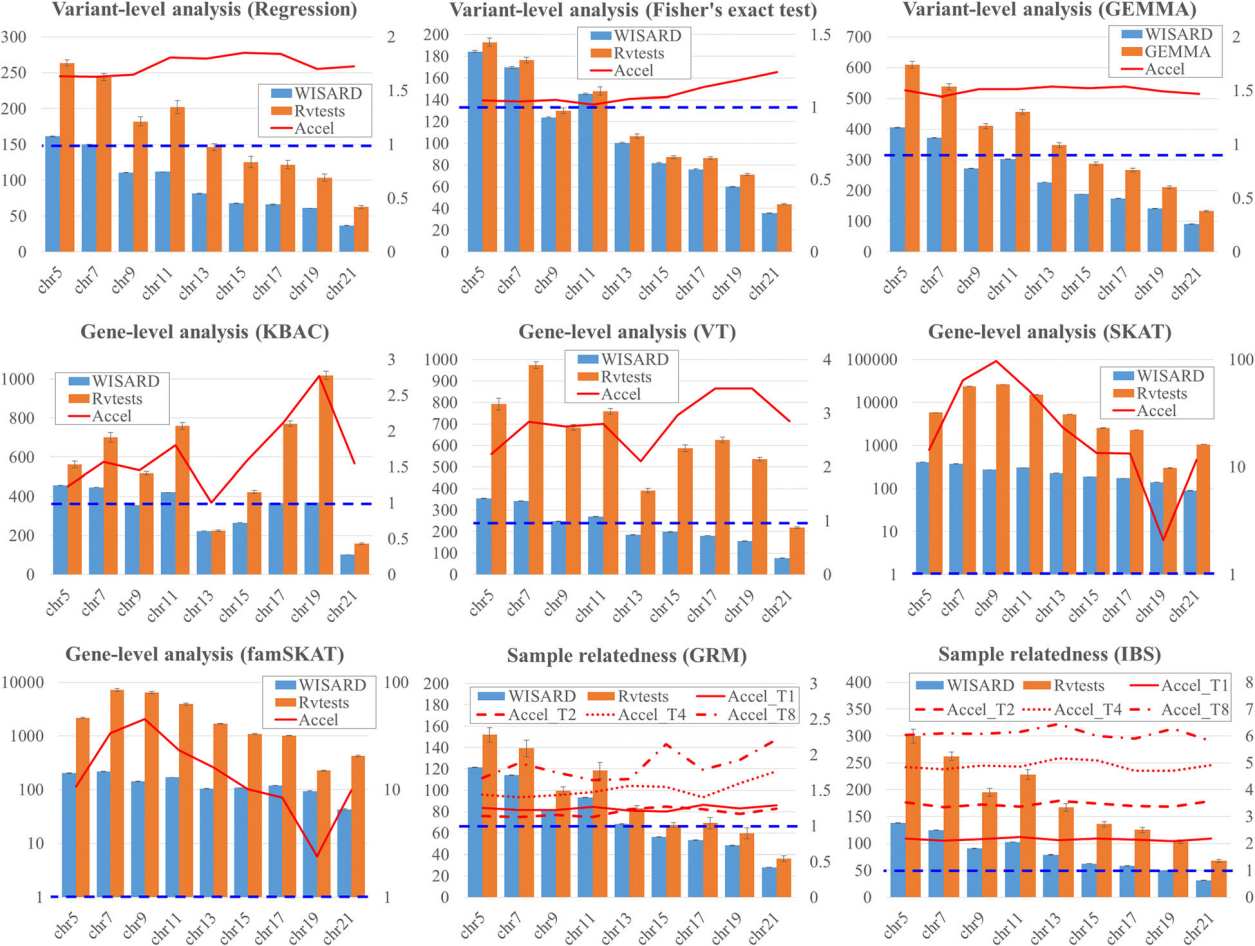
Comparisons of computational time. Computational times were compared with GAW18 simulation data. In each plot, bars indicate execution time in seconds, and their amount can be obtained from left y-axis. Confidence intervals were calculated from five runs. Right y-axis is for red lines and they indicate relative ratios between WISARD and other existing toolset. Relative ratios which are larger than 1 indicate that WISARD is computationally faster, and horizontal blue dashed line indicates 1 for relative ratios. Regression and Fisher’s exact test from WISARD were compared with results from R. In the plots for GRM and IBS, dashed, dotted and dash-dotted red lines indicate relative ratios when 2, 4 and 8 threads of WISARD are used, compared to Rvtests with the same number of threads

Computational efficiency of WISARD was compared with GAW18 dataset. Figure 2 shows that WISARD consistently has superior performance than Rvtests and GEMMA up to twice acceleration (Fig. 2). For variant-level association analyses with linear mixed models, WISARD was compared with GEMMA and was around 1.7 times faster (Fig. 2). Even though GEMMA has been a well-optimized program coded in C/C++ with high-performance matrix calculation library, our implementation achieved further computational improvement. Performance of gene-level analyses with WISARD showed even more differences.

Figure 2 also shows that WISARD outperforms Rvtests in all tests we considered. Largest difference of gene-level analyses was observed for SKAT analyses, and computation with WISARD is 205 times faster. For IBS and GRM calculation, Rvtests use vcf2kinship, and it is used for comparison. Figure 2 shows that WISARD is consistently around 2.3 times faster than Rvtests for IBS calculation, and slightly better for GRM calculation. If two or more threads are used, their differences become larger.

Last we compared results from GAW18 dataset by WISARD and compared toolsets, and check whether their results are same. Additional file 1: Table S1 shows their differences are almost negligible.

### Evaluations of new methods with GAW17 datasets

**Fig. 3:**
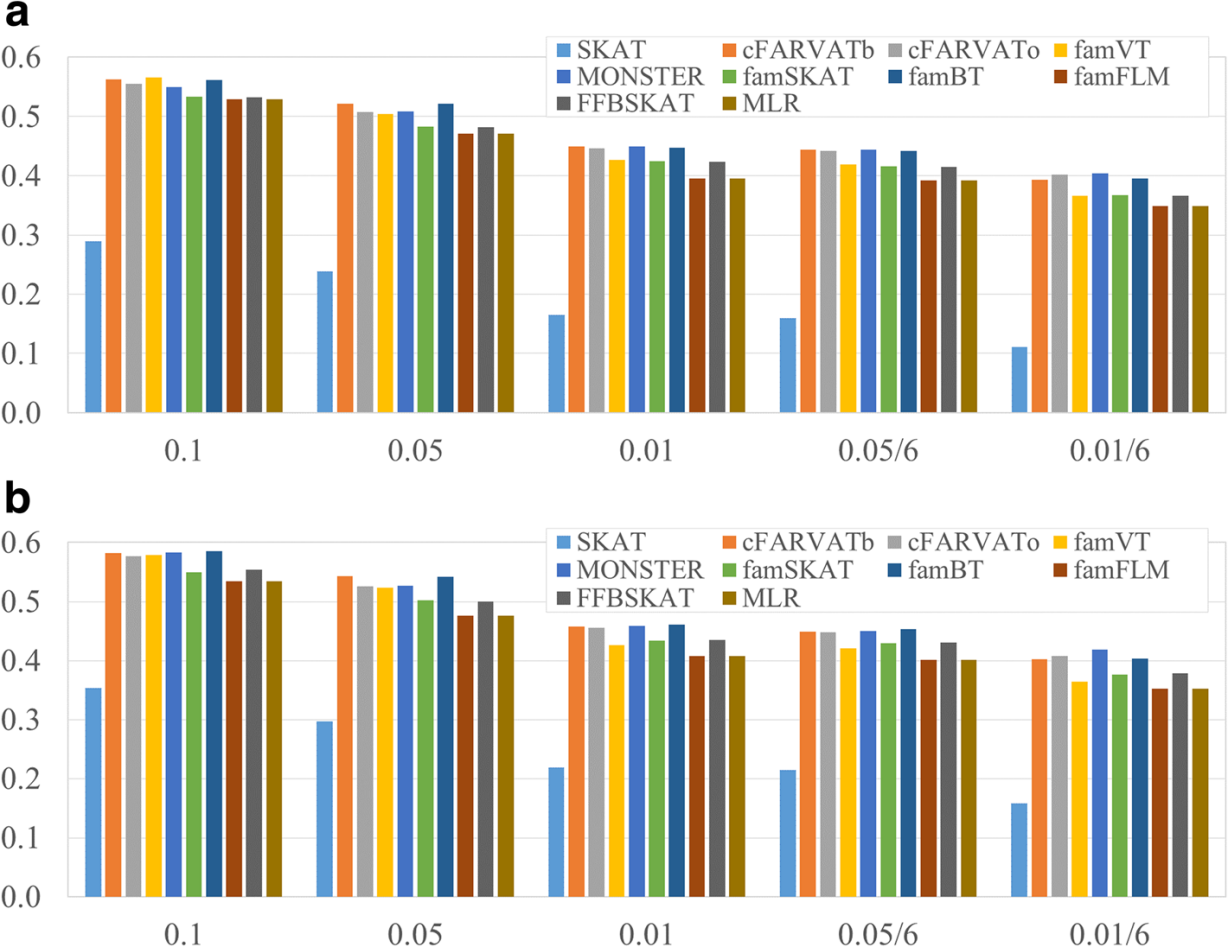
Power comparison of the proposed methods (cFARVAT-b, cFARVAT-o and famVT) and compared methods. Statistical powers were estimated with GAW17 dataset. X and Y axes indicate significance level for power evaluation and statistical power estimates, respectively. Figures (**a**) and (**b**) show results without and with PC adjustment, respectively

We estimated type-1 errors and statistical powers of the proposed methods with GAW17 dataset and they were compared with other methods. Table 3 shows that all methods except MONSTER preserve the nominal type-1 error rates. MONSTER consistently shows inflated type-1 error rates, and it is partially due to the population substructures because it cannot utilize IBS or GRM. Next, we calculated the empirical statistical powers with 200 replicates. Figure 3a and b show the empirical power estimates without and with PC adjustments, and adjustment with PC scores generally improved the statistical power of all methods. PC scores were estimated with EIGENSTRAT approach [39]. cFARVATo always exhibits good performance, and famVT becomes modest at the smaller significance levels. MONSTER has good statistical powers, but does not control the nominal significance level correctly. SKAT and three methods from FREGAT (famFLM, FFBSKAT and MLR) have lower statistical powers than other methods for all scenarios. SKAT showed lowest performance and it may be attributable to misspecified variance-covariance matrix. Therefore we can conclude that the proposed methods have good performance, compared to existing toolsets.

**Table 3:** Estimated type-1 error rates

*α*	WISARD					
	cFARVAT-s	cFARVAT-b		cFARVAT-o	famVT	
0.1	0.093 (±0.024)	0.096 (±0.023)		0.093 (±0.022)	0.081 (±0.02)	
0.05	0.047 (±0.016)	0.048 (±0.017)		0.048 (±0.016)	0.043 (±0.016)	
0.01	0.01 (±0.006)	0.01 (±0.007)		0.011 (±0.007)	0.012 (±0.008)	
*α*	famSKAT	MONSTER	FREGAT			
			famBT	famFLM	FFBSKAT	MLR
0.1	0.097 (±0.029)	0.128 (±0.021)	0.1 (±0.03)	0.104 (±0.032)	0.101 (±0.032)	0.104 (±0.032)
0.05	0.05 (±0.02)	0.072 (±0.019)	0.048 (±0.02)	0.051 (±0.023)	0.058 (±0.022)	0.053 (±0.023)
0.01	0.011 (±0.008)	0.022 (±0.006)	0.01 (±0.008)	0.016 (±0.014)	0.012 (±0.009)	0.016 (±0.013)

Empirical type-1 error rates at the several significance levels and their standard errors which is in parenthesis were estimated with GAW17 simulation data

### Real data analysis of EOCOPD dataset

**Fig. 4:**
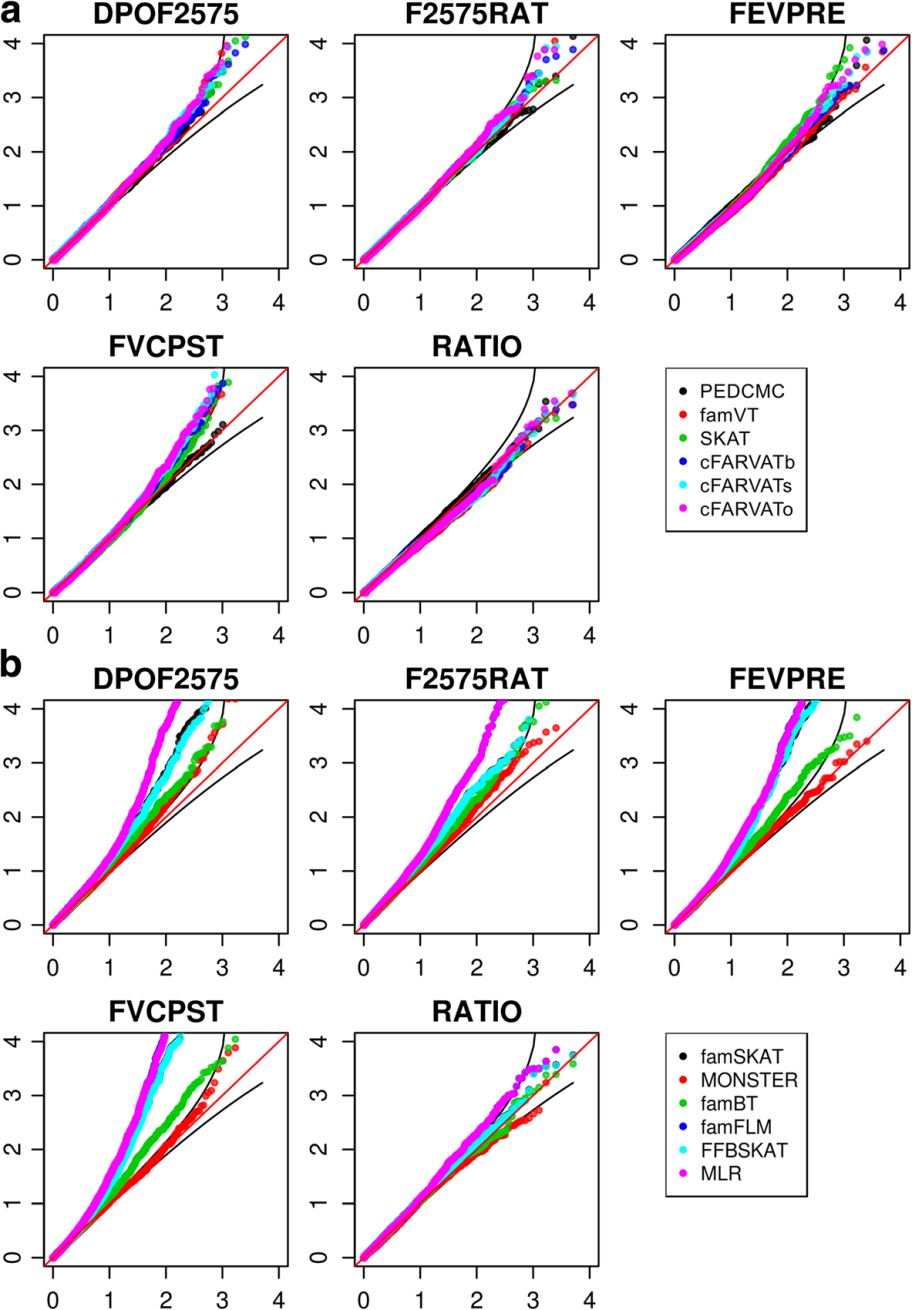
QQ plots of results from WISARD and compared methods for phenotypes of EOCOPD dataset. Red and black straight lines indicate *y* = *x*, and its confidence intervals respectively. **a** QQ plots of results from WISARD methods. **b** QQ plots of results from compared methods

Analyses results for 5 phenotypes of EOCOPD dataset are summarized in Fig. 4. Figure 4a and b indicate quantile-quantile (QQ) plots for all phenotypes with statistics implemented in WISARD and compared toolset respectively. In Fig. 4a, cFARVAT-o and famVT are newly proposed methods, and pedCMC was proposed by Zhu and Xiong [27]. Figure 4 shows that results are quite similar among methods. Rare variant analyses of FVCPST with cFARVAT showed moderate inflation, and results from other phenotypes seem to be statistically valid. Statistics implemented by compared toolsets are generally inflated except MONSTER. MONSTER showed the similar pattern as the proposed methods, but results for DPOF2575 tend to be liberal. In contrast, famSKAT method consistently has inflated *P* values for all phenotypes, which leads to a large number of false positives. Four methods implemented in FREGAT (famBT, famFLM, FFBSKAT, and MLR) consistently showed inflated pattern except for RATIO as well.

Table 4 shows the number of significant genes at the Bonferroni-adjusted 0.05 significance level by the number of analyzed genes. It should be noted that *P* values from famSKAT, famFLM, FREGAT and MLR, tend to be liberal and it is why they have many significant results. WISARD was a unique toolset that preserves the nominal significance level and identified one or more significant genes for all phenotypes. *P* values from MONSTER are generally stable, but it was not able to discover any significant gene except DPOF2575. Thus we focused on genes identified by WISARD due to its unacceptably high Q-Q trend of famSKAT, famFLM, famSKAT and FFBSKAT, and SKAT. Additional file 1: Table S2 shows summary for significant genes by WISARD. According to our results, for DPOF2575 and F2575RAT, our methods except cFARVATb identified FGD5. In addition, association of B3GNTL1 and SLC2A7 for FVCPST were also identified from famVT and 3 cFARVAT, respectively. FGD5 belongs to RhoGEF family, and activates expression of CDC42. For the other genes of RhoGEF family and CDC42, previous investigation revealed their role as a druggable target of COPD [40, 41], as well as their relationship of COPD [42]. SLC2A7 (GLUT7) is a member of glucose transporters (GLUT) family, which shows a substantial relationship with COPD [43]. PRRG2 and CENPQ are newly discovered genes, and further investigation for both are necessary.

**Table 4:** Number of significant genes at the Bonferroni-adjusted 0.05 significance level

Phenotype	WISARD					
	PedCMC	famVT	SKAT	cFARVAT-s	cFARVAT-b	cFARVAT-o
DPOF2575	4	**2**	0	**1**	**0**	**1**
F2575RAT	0	**1**	1	**1**	**0**	**1**
FEVPRE	1	**0**	1	**0**	**0**	**0**
FVCPST	4	**1**	2	**3**	**2**	**3**
RATIO	1	**0**	0	**0**	**0**	**0**
Phenotype	famSKAT	MONSTER	FREGAT			
			famBT	famFLM	FFBSKAT	MLR
DPOF2575	5	1	1	11	5	11
F2575RAT	0	0	0	4	0	4
FEVPRE	2	0	0	11	3	11
FVCPST	9	0	0	34	9	34
RATIO	0	0	0	0	0	0

Rare variant association analyses of DPOF2575, F2575RAT, FEVPRE, FVCPST and RATIO were conducted with EOCOPD data. Upper and lower table display results from WISARD and other toolsets, respectively. Bolded numbers represent the number of identified genes from newly proposed methods

### Summary of analysis results

**Fig. 5:**
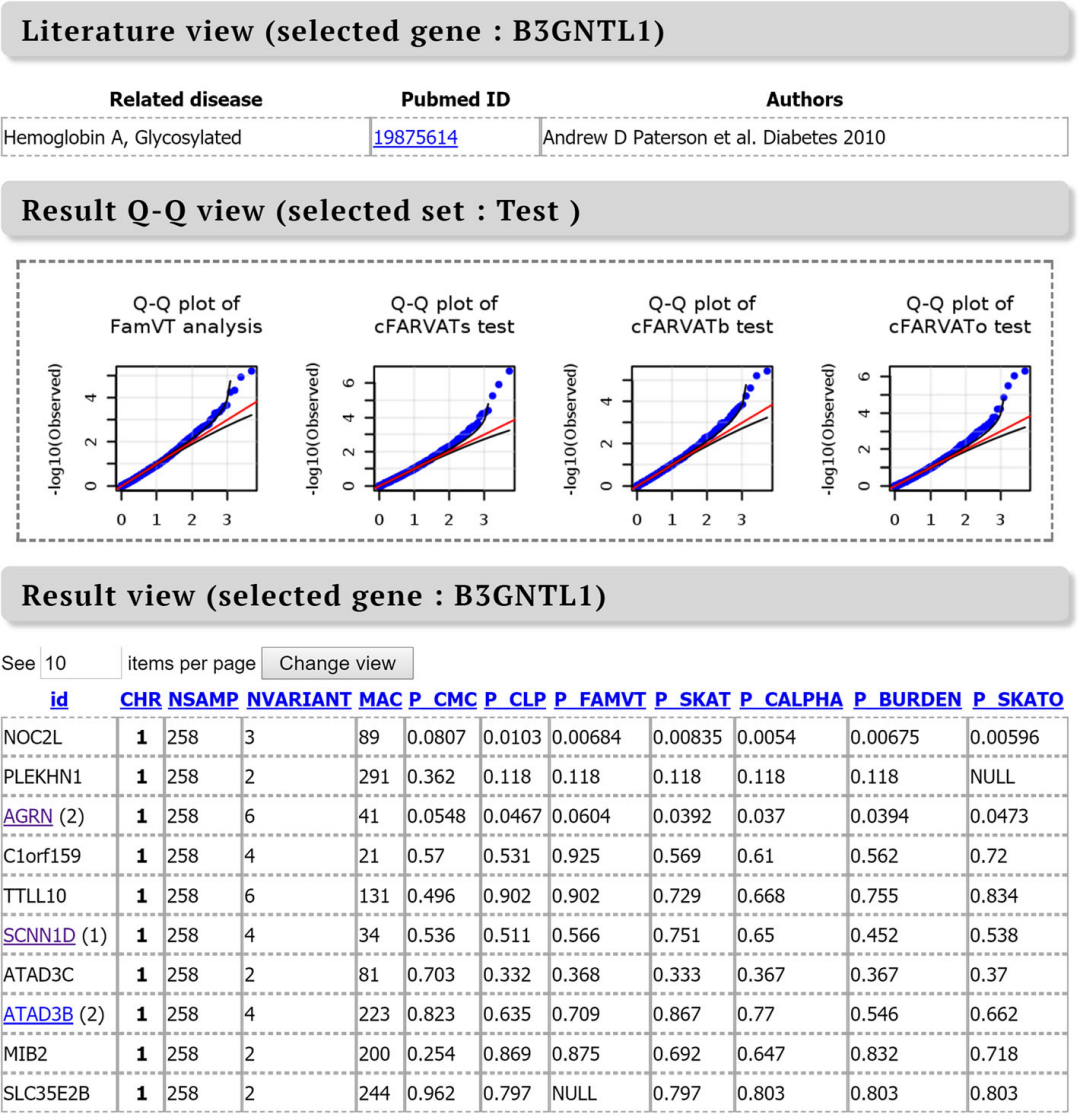
An example of plots and summary tables generated from Web-WISARD

WISARD enables automatic visualization of the results of statistical analysis, using commands in the R program. Furthermore, the web-based WISARD can annotate each marker and provide information about the disease susceptibility loci reported in the GWAS catalogue, the Human Gene Mutation Database (HGMD) and Online Mendelian inheritance in Man (OMIM). Figure 5 depicts the result from web-based WISARD applied to EOCOPD data.

## Discussion

Over the last decade, thousands of GWAS have been conducted to identify disease susceptibility loci, and many causal variants for phenotypes have been identified. However, missing heritability remains a challenge, and genetic analysis with next-generation sequencing technology has been expected to provide some clues. Even though various genetic analyses have been conducted to address these unsolved questions, most of them have not yet been answered, and development of an analysis toolset that enables thorough and comprehensive analysis is in demand.

In this paper, we present a comprehensive workbench, WISARD, for the analysis of large-scale genetic data with family-based samples. WISARD provides various functions for quality control, data management and extensive statistical analyses for family-based samples, and it is also useful for population-based samples in the presence of a population substructure. The quality of each variant and subject can be evaluated with familial relationship, and statistical analyses can be conducted by allowing for phenotypic and genetic correlation between subjects. WISARD takes account of correlations between subjects, and our analysis with simulated data showed that WISARD outperforms similar existing toolsets with respect to computational time, which implies that the genome-wide analysis is achievable in a relatively short time. Furthermore, we proposed two novel methods for rare variant association analyses with related samples, and found that it achieves reasonable statistical power and preserves the nominal significance levels. Moreover, application of the proposed methods to EOCOPD dataset successfully identified significant genes, and thus these results illustrate its practical value.

Recent improvements in genotyping technology enable the identification of rare variants for common diseases, and large families have been expected to play a key role for rare variant association analysis. However, in spite of the various advantages of family-based designs, their statistical analysis has often been complicated because of relatedness between family members. WISARD provides comprehensive functions for various genetic analyses with large families, and it enables researchers’ efficient large-scale genetic analysis.

## Additional file

Additional file 1: Supplementary Text. **Table S1.** Accuracy of WISARD’s implementation. GAW18 dataset was analyzed with WISARD and existing toolset. Then *P*-values from WISARD and existing toolsets were compared, and averages of their differences were obtained. Regression and Fisher’s exact test from WISARD were compared with results from R. **Table S2.** List of significant genes from statistics implemented in WISARD. famVT and cFARVAT-o are newly proposed methods. (Chr = chromosome, # var. = number of variants in the gene, MAC = sum of minor allele count). **Figure S1.** Multithreading efficiency of WISARD analyses with varying number of threads. Acceleration folds of the nine analyses with (A) 2 threads, (B) 4 threads, and (C) 8 threads were obtained. X and Y axes respectively represent chromosomes of GAW18 dataset and acceleration folds compared to the single-thread execution time. Solid lines represent observed acceleration folds of nine different analyses, and red dashed line represents upper limit of speedup with given number of threads. Regression and Fisher tests by WISARD were compared with results by R. (DOCX 138 kb)
