# Supplementary material for: WISARD: workbench for integrated superfast association studies for related datasets
Source: BMC Med Genomics. 2018 Apr 20;11(Suppl 2):39. doi: 10.1186/s12920-018-0345-y (PMC5918457; doi:10.1186/s12920-018-0345-y)

**Additional file 1**

**Table S1. Accuracy of WISARD’s implementation** GAW18 dataset was analyzed with WISARD and existing toolset. Then P-values from WISARD and existing toolsets were compared, and averages of their differences were obtained. Regression and Fisher’s exact test from WISARD were compared with results from R.

| Analysis | Regression (R) | Fisher’s exact test (R) | GEMMA | KBAC | VT | SKAT |
| --- | --- | --- | --- | --- | --- | --- |
| Difference | 0 | 0 | <10^-6^ | 0.00144 | 0.00217 | <10^-5^ |
| Analysis | famSKAT | GRM | IBS |  |  |  |
| Difference | <10^-5^ | <10^-5^ | 0 |  |  |  |

**Table S2. List of significant genes from statistics implemented in WISARD** famVT and cFARVAT-o are newly proposed methods. (Chr = chromosome, # var = number of variants in the gene, MAC = sum of minor allele count)

| **Phenotype** | **Chr** | **Gene** | **# var** | **MAC** | **famVT** | **cFARVAT** | | | **MONSTER** | **famBT** |
| --- | --- | --- | --- | --- | --- | --- | --- | --- | --- | --- |
|  |  |  |  |  |  | **b** | **s** | **o** |  |  |
| **DPOF2575** | 3 | **FGD5** | 3 | 70 | **8.80E-06** | 1.49E-04 | **1.34E-05** | **8.31E-06** | 0.2488319 | 0.471068 |
|  | 8 | SPATC1 | 3 | 26 | **5.53E-06** | 0.347625 | 0.159374 | 0.172966 | 0.0489315 | 0.366053 |
|  | 19 | PRRG2 | 2 | 61 | 0.575176 | 0.572759 | 0.623383 | 0.578796 | **6.13E-06** | 0.536557 |
|  | 6 | CENPQ | 2 | 511 | 0.449522 | 0.448781 | 0.503948 | 0.45201 | 0.3631479 | **2.29E-06** |
| **F2575RAT** | 3 | **FGD5** | 3 | 74 | **8.80E-06** | 1.49E-04 | 1.34E-05 | **8.31E-06** | 0.2488319 | 0.471068 |
| **FVCPST** | 17 | **B3GNTL1** | 2 | 114 | **5.97E-06** | **3.17E-06** | **3.53E-06** | **3.19E-06** | 0.9579092 | 0.000498 |
|  |  | **SLC2A7** | 2 | 71 | 1.13E-05 | **8.26E-06** | **8.99E-07** | **5.92E-06** | 0.2247 | 0.07695 |
|  | 12 | EP400NL | 2 | 4 | 0.001914 | 1.03E-03 | **4.94E-08** | **4.90E-07** | 0.2761631 | 0.000606 |

**Figure S1. Multithreading efficiency of WISARD analyses with varying number of threads.** Acceleration folds of the nine analyses with (A) 2 threads, (B) 4 threads, and (C) 8 threads were obtained. X and Y axes respectively represent chromosomes of GAW18 dataset and acceleration folds compared to the single-thread execution time. Solid lines represent observed acceleration folds of nine different analyses, and red dashed line represents upper limit of speedup with given number of threads. Regression and Fisher tests by WISARD were compared with results by R.


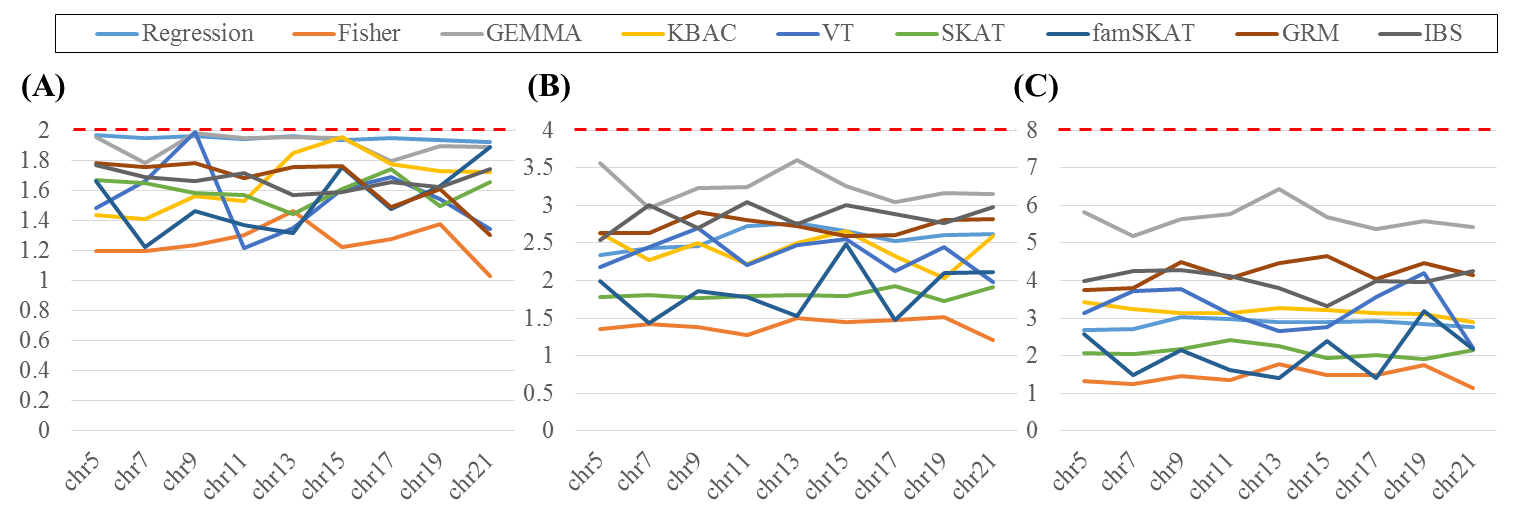

Supplement: Supplementary file 1 — Supplementary Text. Table S1. Accuracy of WISARD’s implementation. GAW18 dataset was analyzed with WISARD and existing toolset. Then P-values from WISARD and existing toolsets were compared, and averages of their differences were obtained. Regression and Fisher’s exact test from WISARD were compared with results from R. Table S2. List of significant genes from statistics implemented in WISARD. famVT and cFARVAT-o are newly proposed methods. (Chr = chromosome, # var. = number of variants in the gene, MAC = sum of minor allele count). Figure S1. Multithreading efficiency of WISARD analyses with varying number of threads. Acceleration folds of the nine analyses with (A) 2 threads, (B) 4 threads, and (C) 8 threads were obtained. X and Y axes respectively represent chromosomes of GAW18 dataset and acceleration folds compared to the single-thread execution time. Solid lines represent observed acceleration folds of nine different analyses, and red dashed line represents upper limit of speedup with given number of threads. Regression and Fisher tests by WISARD were compared with results by R. (DOCX 138 kb) [file 12920_2018_345_MOESM1_ESM.docx]
